# Alternating Flow Field Design Improves the Performance of Proton Exchange Membrane Fuel Cells

**DOI:** 10.1002/advs.202205305

**Published:** 2022-12-05

**Authors:** Zhengguo Qin, Wenming Huo, Zhiming Bao, Chasen Tongsh, Bowen Wang, Qing Du, Kui Jiao

**Affiliations:** ^1^ State Key Laboratory of Engines Tianjin University Tianjin 300350 China; ^2^ National Industry‐Education Platform of Energy Storage Tianjin University Tianjin 300350 China; ^3^ Department of Building and Real Estate Research Institute for Sustainable Urban Development (RISUD) Research Institute for Smart Energy (RISE) The Hong Kong Polytechnic University Hung Hom, Kowloon Hong Kong 999077 China

**Keywords:** alternating design, flow field, mass transfer, PEMFC, performance

## Abstract

The flow field structure of a proton exchange membrane fuel cell (PEMFC) is a determining factor for improving the cell power density. In this study, a universal alternating flow field design for the first time is proposed, which arranges structural units with different flow resistances in an alternating way to significantly improve the gas transfer rate into the electrode, with the advantages of easy machining and low pumping loss. Based on the design, it is proposed and tested large‐scale fuel cells with three novel flow fields by combining a parallel channel, baffled channel, serpentine channel, and narrowed channel. The results show that the design can significantly enhance the gas supply efficiency and that the novel baffled flow field improves the PEMFC performance by 23% with low pumping loss. The design employed in the study offers additional options for flow field optimization and contributes to the early achievement of next‐generation ultrahigh power density fuel cells.

## Graphical abstract

This work proposes a universal guideline for the flow field design, which significantly improves the gas transfer rate into the electrode. The universal design concept can not only facilitate the proposal of easily processed, optimized flow fields but also enable the further reduction of experimental costs for the design of flow fields of PEMFCs.

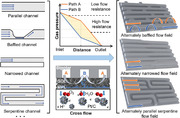

## 1 Introduction

In recent years, reducing the consumption of fossil energy and developing green renewable energy have been essential for the conversion of the worldwide energy structure.^[^ ^1^, ^2^ ^]^ With the rapid development of proton exchange membrane fuel cell (PEMFC) technology, there has been increasing demand for clean and sustainable global energy applications.^[^ ^3^ ^]^ According to Japan's New Energy and Industrial Technology Development Organization (Japan NEDO), further improvements in the stack power density to 6.0 kW L^−1^ and 9.0 kW L^−1^ will inevitably introduce additional technical challenges and will still require an additional ≈20% contribution of power density from flow field plate innovation,^[^ ^4^, ^5^, ^6^ ^]^ which requires continuous modification of the flow field design.

An excellent flow field design can significantly contribute to the increase in the electrochemical reaction rate and improvement in the water and heat management capacity of the PEMFC.^[^ ^7^, ^8^, ^9^ ^]^ To avoid the shortcomings of conventional flow fields,^[^ ^10^ ^]^ Liu et al.^[^ ^11^ ^]^ set up a microdistributor at the inlet area of the parallel flow field to improve the uniformity of the intake gas. Youcef et al.^[^ ^12^ ^]^ suggested channel‐to‐rib width ratios for different flow fields to improve cell performance. Wang^[^ ^13^ ^]^ introduced a dot matrix and sloping baffle flow field to strengthen the mass transfer and water removal capacity of a PEMFC. Novel flow fields are also constantly being developed. Seyed^[^ ^14^ ^]^ proposed a honeycomb flow field to decrease the possibility of hotspots and flooding phenomena in PEMFCs. Trogadas et al.^[^ ^15^ ^]^ employed a lung‐inspired flow field to overcome reactant homogeneity issues in PEMFCs. Metal foam functions as an alternative gas distributor for PEMFCs. This material shows better uniformity and convection of the gas reactant due to the divergent transport mode.^[^ ^16^, ^17^, ^18^ ^]^ 3‐D flow fields have gained considerable attention because of their excellent mass transfer to electrodes.^[^ ^19^, ^20^, ^21^ ^]^ At the same time, various tools have been applied to the study of two‐phase flow in fuel cells, such as the volume of fluid method,^[^ ^22^ ^]^ the lattice Boltzmann method,^[^ ^23^ ^]^ and artificial intelligence techniques,^[^ ^24^ ^]^ which provide guidance for the design of the flow field. While a few studies have been conducted on the modification of cathode flow fields, most of them were carried out on a small fuel cell whose mass transfer loss was not obvious. To date, research on flow fields that can generate 3‐D flows has been mostly conducted by simulation because of high manufacturing difficulties.

In this article, we propose a universal alternating design in a flow field, which arranges structural units with different flow resistances in an alternating way to significantly improve the gas concentration at electrodes with a simple manufacturing process and low pumping loss, and apply it to parallel and serpentine flow fields of 106.2 cm^2^ Based on this concept, an alternately baffled flow field, alternately narrowed flow field, and alternately parallel serpentine flow field are designed for cathode air distribution. Test techniques including a constant current polarization curve test (*I*–*V*), electrochemical impedance spectroscopy (EIS), and pressure drop measurement are used to identify PEMFC performance under different operation conditions, meanwhile, a computational fluid dynamics (CFD) model of a PEMFC is adopted to analyze the internal transport of a PEMFC with different flow fields.^[^ ^25^, ^26^ ^]^

## 2 Results and Discussion

### 2.1 Comparison of the Flow Fields Based on the Alternating Design

**Figure 1:**
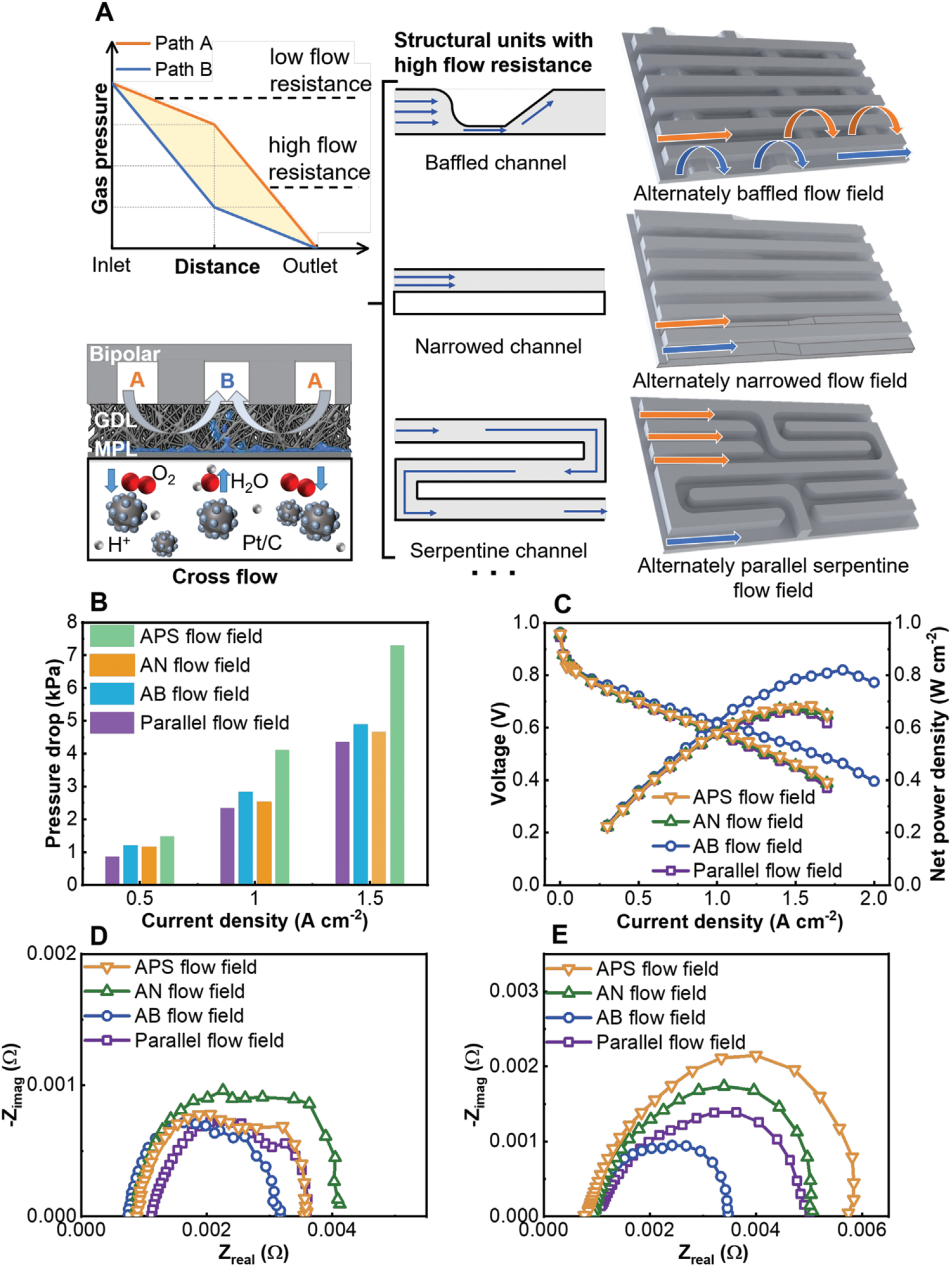
Comparison of the flow fields based on the alternating design. A) Schematic of the alternating design and 3D structure diagram of novel flow fields. Experimental comparison of the B) pressure drop; C) polarization curve and net power density; D) EIS test result under 0.5 A cm^−2^; and E) EIS test result under 1.5 A cm^−2^ among the four cathode flow field designs. Operating conditions (anode/cathode): RH (100%/40%), ST (1.5/2.5), outlet back pressure (0 kPa/0 kPa), operating temperature (343.15 K).

To achieve efficient mass transfer to the electrode, we propose a concept of universal alternating design, as shown in **Figure** **1**A. There are two structural units in each path of the flow field. When the gas flows through the flow field, one structural unit with a higher flow resistance will create a higher pressure drop, while the other structural unit has a lower pressure drop. The two types of structural units are arranged alternately in the flow field. The amount of flow resistance is indicated by the slope of the pressure‐distance curve. The pressure difference between adjacent paths is created by the alternating design, which is also observed by the simulation results in Figure S1, Supporting Information. For each gas flow path, the type and number of structural units are the same to ensure equal flow resistance. Gas convection in the under‐ridge area, also called cross flow, is created by the pressure difference between adjacent paths. This process has been proven beneficial for mass transfer and liquid removal in electrodes.

Based on the alternating design, we propose three flow fields with different structural units, as shown in Figure 1A, to optimize the parallel and serpentine flow fields. Compared to the parallel flow channel, gas flows through the baffled channel, the narrowed channel, and the serpentine channel with a higher pressure drop. These structural units and parallel channels are arranged in an alternating way. The three modified flow fields are named according to the added structural units, that is, alternately baffled (AB) flow field, alternately narrowed (AN) flow field, and alternately parallel serpentine (APS) flow field. The structural parameters and the complete structure of various flow fields are shown in Table S1 and Figure S2, Supporting Information. For the AB flow field, the shape of the baffle corresponds to Chen's design.^[^ ^27^, ^28^, ^29^ ^]^

The performance of a PEMFC with various cathode flow fields is compared under a cathode relative humidity (RH_c_) of 40% and cathode stoichiometric ratio (ST_c_) of 2.5. Figure 1B,C shows that the addition of baffles or narrowed channels increases the overall pressure drop of the flow field, but it is negligible. The addition of alternately serpentine channels largely improves the pressure drop because of the high flow resistance. The maximum net power density of the PEMFC with the AB flow field is 0.82 W cm^−2^, which is 23% higher than that with the parallel flow field and is achieved at a larger operation current density. However, the introduction of serpentine channels and narrowed channels fails to boost the cell performance compared with the parallel flow field (with a maximum net power density of 0.667 W cm^−2^), whose maximum net power density is 0.684 W cm^−2^ and 0.674 W cm^−2^, respectively. An experimental comparison of the power density among the different cathode flow field designs is shown in **Table** **1**.

**Table 1:** Comparison of the power density among the different cathode flow field designs under RH_c_ 40% and ST_c_ 2.5

Flow field	Parallel	AB	AN	APS	Full baffled	EAB
The maximum net power density (W cm^−2^)	0.667	0.820	0.674	0.684	0.724	0.668
Corresponding current density (A cm^−2^)	1.5	1.8	1.5	1.6	1.6	1.6

As shown in Figure 1D,E. The ohmic resistance of the three novel plates is less than the original parallel flow field under both low and high current density conditions. This can be attributed to the effects of cross flow at the channel/gas diffusion layer (GDL) interface induced by the unique structure of the alternating design.^[^ ^30^ ^]^ The humidified gas is blown into the GDL by cross flow and humidifies the membrane, which improves the proton conductivity and reduces the overall ohmic loss. The internal transfer of PEMFCs is researched by the CFD model built in Figure S3–S4, Supporting Information.

In addition to the cross flow, the featured structure of baffles forces the air to enter the GDL and improves the convection transport in the GDL, which makes the AB flow field capable of removing the accumulated liquid water under the lands.^[^ ^31^, ^32^ ^]^ This helps to relieve the blockage effect of liquid on oxygen transport to the electro‐chemically active sites, as shown in Figure S5–S6, Supporting Information. However, the alternating designs with applied narrowed channels and serpentine channels show large mass transfer resistance. For the AN flow field, as a result of its reduced height/width ratio of channels, the oxygen concentration distribution is uneven, and oxygen starvation occurs in localized areas, as shown in Figure S7, Supporting Information, which increases the overall activation and mass transfer loss.^[^ ^33^ ^]^ For the APS flow field, water tends to accumulate in the turns of serpentine channels and hinders oxygen transport. Oxygen starvation may occur in the downstream areas due to the increased length of the flowing path of serpentine channels. Optimization of turn‐in serpentine channels can improve its water removal capacity.^[^ ^34^, ^35^ ^]^ However, a larger air flow rate is found to alleviate the above problems of these two new flow fields to improve the performance of the PEMFC, as shown in Figure S8–S10, Supporting Information.

### 2.2 Performance of the Alternately Baffled Flow Field Under Different Air Intake Conditions

**Figure 2:**
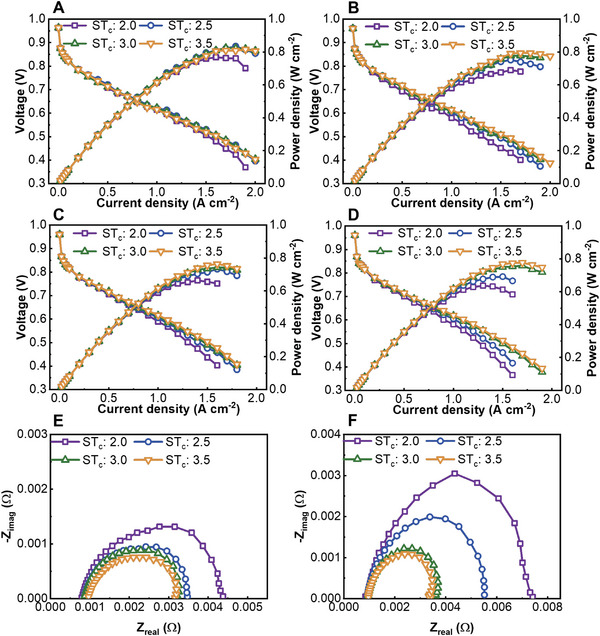
The experimental polarization curve of the PEMFC using the cathode AB flow field with an RH of cathode inlet air of A) 40%, B) 60%, C) 80%, and D) 100%. The EIS test result of the PEMFC using the cathode AB flow field under 1.5 A cm^−2^: E) RH_c_ 40% and (F) RH_c_ 100%.

The sensitivity of the alternating design to changes under air operating conditions is discussed in this section. The same anode operating condition and cell outlet back pressure as the other cases were used. The test results of the PEMFC under different RH_c_ and ST_c_ values are shown in **Figure** **2**A–D. For a flow field containing an alternating design, the mismatch between the flow rate and the RH of the intake air will cause a negative effect on the performance of the PEMFC. As shown in Figure 2A,E, when the RH of the intake air is 40%, excessive air purges the moisture out from the membrane electrode assembly (MEA), and the ohmic resistance increases with a reduced hydration degree of the membrane. If the flow rate of the inlet air is too low, then the water droplets accumulate and increase the mass transfer loss of air. When the ST_c_ of the intake air is 2.5, the intake air with an RH of 40% will be blown into the MEA by cross flow to achieve a better hydration degree and maintain low ohmic resistance. During the operating conditions, the water produced by the reaction can also be discharged in time to prevent excessive mass transfer loss and activation loss.

According to the *I*–*V* curves in Figure 2B–D, when the RH of the intake air is high, a large intake air flow rate is required to remove water and then lower the activation and concentration loss. There is no significant ohmic impedance difference for the PEMFC among different ST_c_ values, as shown in Figure 2F, which indicates the small effect of ST_c_ on the hydration degree of the membrane under a high RH of intake air. The performance is mainly determined by mass transfer loss and activation loss when intake air is at a high RH.

### 2.3 Extended Application of the Alternating Design

**Figure 3:**
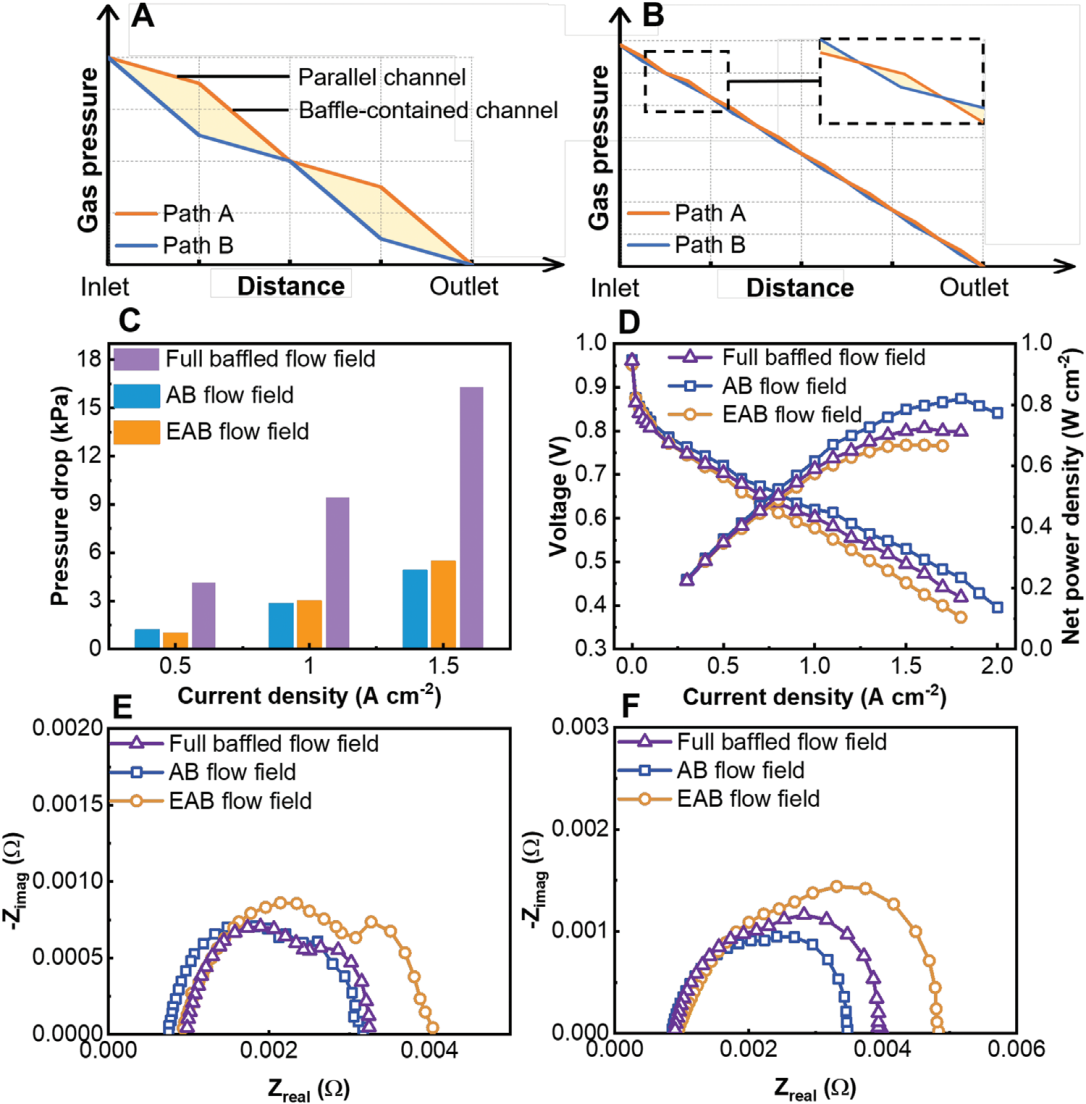
Comparison of parallel flow fields with different baffle arrangements. Schematic of extended applications of the alternating design in A) EAB flow field and B) full baffled flow field. Experimental comparison of the C) pressure drop; D) polarization curve and net power density; E) EIS test result under 0.5 A cm^−2^; and F) EIS test result under 1.5 A cm^−2^ among the three cathode flow field designs. Operating conditions (anode/cathode): RH (100%/40%), ST (1.5/2.5), outlet back pressure (0 kPa/0 kPa), and operating temperature (343.15 K).

In this section, we present an extended application of the alternating design. As shown in **Figure** **3**A, the extended alternately baffled (EAB) flow field is proposed. The complete flow field is shown in Figure S1‐S2, Supporting Information. Although the number of baffles in channels is the same as that in the AB flow field, the main flow field is divided into four parts along the flow direction. There are four structural units in each path of the flow field. The shorter structural units will lead to a lower pressure difference between adjacent channels. The intensity and location of the air cross flow are changed. As shown in Figure 3B, the full baffled flow field is proposed when the baffle‐free channels of the AB flow field are filled by baffles in the same interval and shape. This is the conventional arrangement of the baffled flow field.^[^ ^36^ ^]^ There is a negligible pressure difference between adjacent paths.

As shown in Figure 3C,D, although the baffles of the full baffled flow field increase the air transmission in the through‐plane direction, the pumping loss is therefore greatly increased. The maximum net power density of the PEMFC with the AB flow field is 13.3% higher than that of the PEMFC with the full baffled flow field. It is achieved at a higher current density than others. This shows that the alternating design can indeed achieve efficient mass transfer by using fewer baffles. Fewer baffles reduce manufacturing costs, especially for graphite. The EAB flow field shows low performance because of its weaker cross flow. The performances of PEMFCs using the EAB flow field and full baffled flow field under various cathode operating conditions are shown in Figures S11 and S12, Supporting Information.

From the EIS test in Figure 3E,F, the ohmic impedance of the PEMFC with an AB flow field is always small even at a low current density because of its excellent water management capacity. For a full baffled flow field, there is not such a pressure difference between adjacent channels, and the high performance of a PEMFC assembled with a full baffled flow field depends on the forcing gas flow in the direction of the through‐plane by multiple baffles but not the cross flow. The EAB flow field has neither a strong cross flow nor enough baffles to force gas flow. Activation loss and mass transfer loss are increased due to its weaker water management capacity.

It should be noted that excessive cross flow will purge the water used to ensure the hydration degree of the membrane, such as the interdigital flow field.^[^ ^37^ ^]^ The intensity and location of the cross flow in our design are related to the type, length, and number of structural units in the flow path. Therefore, although the EAB flow field does not significantly improve the performance of a PEMFC with an active area of 106.2 cm^2^ the arrangement of structural units may create performance improvement in a larger flow field, as shown in Figure S13, Supporting Information.

### 2.4 Application Prospects of the Novel Cathode Flow Field

**Figure 4:**
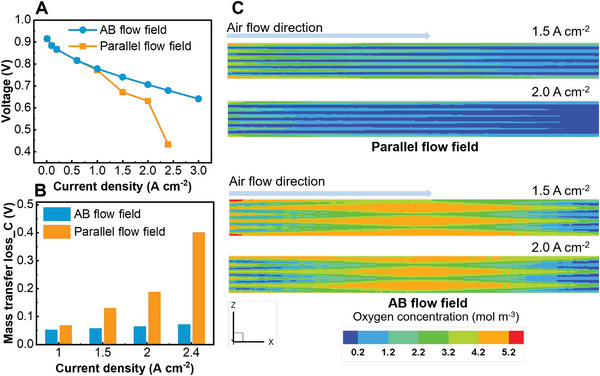
Performance improvements by the novel flow field in the ideal case. Simulated comparison of the (A) polarization curve; (B) mass transfer loss; (C) oxygen concentration in the cathode CL between two flow field designs. Operating conditions (anode/cathode): RH (100%/40%), ST (1.5/2.5), outlet back pressure (0 kPa/0 kPa), and operating temperature (343.15 K)

Based on the simulation model, we assume the case with the assembly pressure and MEA behaving well to study the performance of the AB flow field. The computational domain of the CFD model is shown in Figure S3, Supporting Information. As shown in **Figure** **4**A,B, the performance improvement brought by the alternating baffle arrangement is more significant in this case, especially at high current densities. The performance difference between flow fields is mainly in the mass transfer loss, and the higher the current density is, the more significant the difference. This comparison demonstrates the superior water management capability of the alternating design, which results from the 3D flow of the gas in the flow field.

As shown in Figure 4C, oxygen starvation occurred in the parallel flow field at a high current density. However, the alternating design establishes a pressure difference between adjacent flow channels, and the generated cross flow increases the oxygen concentration of catalyst layer (CL). This reduces the overall activation and concentration loss of the PEMFC, especially when the current density is high. This means that the AB flow field can meet the growing expectations of gas supply for PEMFCs. The comparison demonstrates the potential of an alternating design in the development of future fuel cell flow fields. It contributes to the early achievement of the 2030 power density target set by Japan NEDO.^[^ ^5^ ^]^

## 3 Conclusion

This paper proposed a universal alternating design for the first time and applied it to optimize the cathode flow field in a PEMFC. The design concept intends to enhance the gas mass transfer of a PEMFC by arranging structural units with different flow resistances in an alternating way. Based on this concept, we have proposed several easy‐to‐process, large‐scale flow field designs, all of which have achieved improved performance with low pumping losses. The type and arrangement of structural units in the alternating design and its applicable operating conditions are studied. The main conclusions are as follows:

(1) The alternating design is universal, and multiple novel flow fields based on it show good performance. Furthermore, the water management of the flow field added by alternating baffles is superior to the other two flow fields due to cross flow combined gas flow in the through‐plane direction forced by baffles. The pumping losses of the flow field with alternating baffles and alternately narrowed channels are close to the conventional parallel flow field.
(2) The maximum net power of the PEMFC with a cathode AB flow field is 23% higher than that of the cell with a cathode parallel flow field and 13.3% higher than that of the cell with a conventional baffled flow field. Meanwhile, the pumping loss of the AB flow field is also much lower than that of the conventional baffled flow field.
(3) The intensity and location of cross flow can be adjusted by changing the type and arrangement of structural units in the alternating design to create effective mass transfer to electrodes.
(4) Under different operating conditions, the PEMFC with an AB flow field performs the best under an RH_c_ of 40% and ST_c_ of 2.5. For a cathode flow field containing an alternating design, the mismatch between the intake air flow rate and the RH will cause a negative effect on the performance of the PEMFC.
(5) The alternating design can effectively increase the gas concentration in the CL. There is no oxygen starvation for a PEMFC with a cathode AB flow field even at high current densities, which shows its excellent application potential.

## 4 Experimental Section

### Materials and Sizes of the Fuel Cells

The experimental system settings and related components were shown in Figure S14–S15, Supporting Information. In this study, the material of the end plate was aluminum alloy with an area of 285 × 101 mm^2^ and a thickness of 18 mm. The current collector plate was made of highly conductive gold‐plated copper with an area of 261 × 77 mm^2^ and a thickness of 1.5 mm. The flow field plate in the experiments was made of a graphite plate (Jiangsu Shenzhou Carbon Co., Ltd.) with an area of 261 × 77 mm^2^ and thickness of 4.8 mm, and the flow field machined on the plate had an area of 180 × 59 mm^2^. The flow fields presented in this paper were easily manufactured. Though graphite was used as the flow field plate material in this study, other materials could also be manufactured as the flow field plate, such as composite and metal materials. Manufacturing techniques such as cutting, injection molding, and stamping were mature enough to process these materials.^[^ ^38^, ^39^ ^]^ The active area of the PEMFC was 180 × 59 mm^2^ . For the MEA (Wuhan WUT HyPower Technology Co., Ltd.), the platinum loadings in the CLs of cathode and anode were 0.4 mg cm^−2^ and 0.1 mg cm^−2^, respectively. Both the anode and cathode carbon papers were manufactured with a microporous layer (MPL).

### Fuel Cell Testing

The PEMFCs were assembled at a pressure of 3.5 kN with the help of a universal testing machine (Changchun Kexin Testing Instrument Co., Ltd.). After assembly, the gas pressure of cathode and anode inlet was set 100 kPa. If the pressure decrease did not exceed 0.1 kPa after 5 min, the gas tightness was considered acceptable. A Peltier temperature control system (Wuhan Tailunte Century Technology Co., Ltd) was installed on both end plates of the PEMFC to control the operating temperature with an accuracy of ±1.7 °C.

A commercial fuel cell test station (Ningbo Bate Technology Co., Ltd.) was connected to the experimental PEMFC, which controls the flow rate, temperature, humidity of the reactant gases, and electric load. With cooperation from an electrochemical workstation (Zahner Zennium pro), polarization curve and EIS tests were carried out. The outlet back pressure of the anode and cathode remained at 0 kPa. The anode operating conditions (RH_a_ = 100%, ST_a_ = 1.5) and PEMFC operating temperature (343.15 K) were kept constant in all experiments. PEMFCs with various kinds of cathode flow fields were tested under different cathode operating conditions. The RH_c_ was set as 40%, 60%, 80%, and 100%; the ST_c_ was set as 2.0, 2.5, 3.0, and 3.5. The raw power density (*E_raw_*) produced by the fuel cell is simply the current density, *i*, multiplied by the cell voltage, *V*. The net power of the PEMFC is expressed as follows.^[^ ^40^ ^]^

(1) $$E_{\mathrm{net}}=E_{\mathrm{raw}}\;-E_{\mathrm{pump}}$$

(2) $$E_{\mathrm{raw}}=\;V.i$$

(3) $$E_{\mathrm{pump}}=\frac{m_{air}C_{p}T}{A\zeta }\;\left[{\left(\frac{P_{\mathrm{inlet}}}{P_{\mathrm{atm}}}\right)}^{\frac{k-1}{k}}-1\right]$$

where *m*_air_ is the mass flow rate of air (kg s^−1^), *C*_p_ is the specific heat of air (J kg^−1^ K^−1^), *T* is the absolute temperature of the air (K), *A* is the active area of the cell (cm^2^), *ζ* is the efficiency of the compressor, *P*_inlet_ is the cathode inlet pumping pressure of the air, *P*_atm_ is atmospheric pressure, and *k* is the specific heat ratio. In most fuel cell vehicles, the air compressor was a twin‐screw rotary compressor. These systems had isentropic efficiencies of ≈0.7.^[^ ^41^, ^42^ ^]^

### Model Description

In this study, a three‐dimensional + one‐dimensional (3D+1D) whole PEMFC model was developed to investigate the configurations of flow fields. As shown in Figure S3, Supporting Information, the 3D domain contains anode and cathode flow channels, GDLs, and MPLs. CLs and membranes were simplified as computational nodes into 1D along the through‐plane direction. These two parts were bridged through two extra layers (ELs) embedded into the 3D domain, in which data were stored and exchanged. Mass, momentum, gas species, energy, liquid water saturation, liquid pressure, and electric potential conservation equations were solved in a 3D model and then provide boundary conditions for the 1D model. In the 1D model, each computational node was set at the component interface, and flux conservation equations were solved to obtain key parameters such as membrane water content, electrochemical reaction rate, ionic potential, and so on. The scalars were transformed into source terms to be incorporated into the conservation equations solved in the 3D domain. Compared with conventional 3D cell models, the 3D+1D model improves calculation efficiency and stability. The model validation was shown in Figure S4, Supporting Information. The model was strictly validated in the previous studies. The involved conversation equations were briefly represented as follows^[^ ^25^, ^26^ ^]^:

Mass:

(4) $$\nabla .\left(\rho_{g}{\vec{u}}_{g}\right)=S_{m}$$

Momentum:

(5) $$\begin{array}{ccc} \nabla .\left(\rho_{g}{\vec{u}}_{g}{\vec{u}}_{g}\right) & = & -\nabla P_{g}+\nabla .\left(\mu_{\mathrm{mix}}\left(\nabla {\vec{u}}_{g}+\nabla {{\vec{u}}_{g}}^{T}\right)-\frac{2}{3}\mu_{\mathrm{mix}}\nabla {\vec{u}}_{g}\right)+S_{u} \end{array}$$

Species:

(6) $$\nabla .\left(\rho_{g}{\vec{u}}_{g}Y_{i}\right)=\nabla .\left(\rho_{g}D_{i,\mathrm{eff}}\nabla Y_{i}\right)+S_{i}$$

Energy:

(7) $$\nabla .\left(\rho_{g}C_{\mathrm{pg},\mathrm{eff}}{\vec{u}}_{g}T\right)=\nabla .\left(k_{\mathrm{eff}}\nabla T\right)+S_{T}$$

Liquid water saturation in the flow channel:

(8) $$\nabla .\left(\rho_{\mathrm{lw}}{\vec{u}}_{\mathrm{lw}}s\right)=\nabla .\left(\rho_{\mathrm{lw}}D_{\mathrm{lw}}\nabla s\right)+S_{\mathrm{lw}}$$

Liquid pressure in porous electrodes:

(9) $$0=\nabla .\left(\rho_{\mathrm{lw}}\frac{Kk_{\mathrm{lw}}}{\mu_{\mathrm{lw}}}\nabla P_{\mathrm{lw}}\right)+S_{\mathrm{lw}}$$

Electronic potential:

(10) $$0=\nabla .\left(\kappa_{\mathrm{ele},\mathrm{eff}}\nabla \phi_{\mathrm{ele}}\right)+S_{\mathrm{ele}}$$

Buter–Volmer equation with agglomerate correction:

(11) $$\begin{array}{ccc} j_{a} & = & i_{0,a}^{\mathrm{ref}}A_{\mathrm{pt}}^{\mathrm{eff}}\theta_{T,a}{\left(\frac{\mathrm{RT}C_{H_{2}}}{H_{H_{2}}C_{H_{2}}^{\mathrm{ref}}}\right)}^{0.5}\\ & & \times \left[\exp\left(\frac{2F\alpha_{a}\eta_{a}}{\mathrm{RT}}\right)-\exp\left(-\frac{2F\left(1-\alpha_{a}\right)\eta_{a}}{\mathrm{RT}}\right)\right] \end{array}$$

(12) $$\begin{array}{ccc} j_{c} & = & \frac{\mathrm{RT}C_{O_{2}}}{H_{O_{2}}}\\ & & \times \;{\left(\frac{C_{O_{2}}^{\mathrm{ref}}}{i_{0,c}^{\mathrm{ref}}A_{\mathrm{pt}}^{\mathrm{eff}}\theta_{T,c}\left[\exp\left(\frac{4F\alpha_{c}\eta_{c}}{\mathrm{RT}}\right)-\exp\left(-\frac{4F\left(1-\alpha_{c}\right)\eta_{c}}{\mathrm{RT}}\right)\right]}+\frac{R_{\mathrm{local}}}{4FA_{\mathrm{im}}}\right)}^{-1} \end{array}$$

## Conflict of Interest

The authors declare no conflict of interest.

## Supporting information

Supporting Information

## Data Availability

Research data are not shared.
