# Supplementary material for: Alternating Flow Field Design Improves the Performance of Proton Exchange Membrane Fuel Cells
Source: Adv Sci (Weinh). 2022 Dec 5;10(4):2205305. doi: 10.1002/advs.202205305 (PMC9896037; doi:10.1002/advs.202205305)
Supplement: Supplementary file 1 — Supporting Information [file ADVS-10-2205305-s001.pdf]

ADVANCED SCIENCE
Open Access

Supporting Information

for *Adv. Sci.*, DOI 10.1002/advs.202205305

Alternating Flow Field Design Improves the Performance of Proton Exchange Membrane Fuel Cells

Zhengguo Qin, Wenming Huo, Zhiming Bao, Chasen Tongsh, Bowen Wang, Qing Du and Kui Jiao**

Supplementary Information

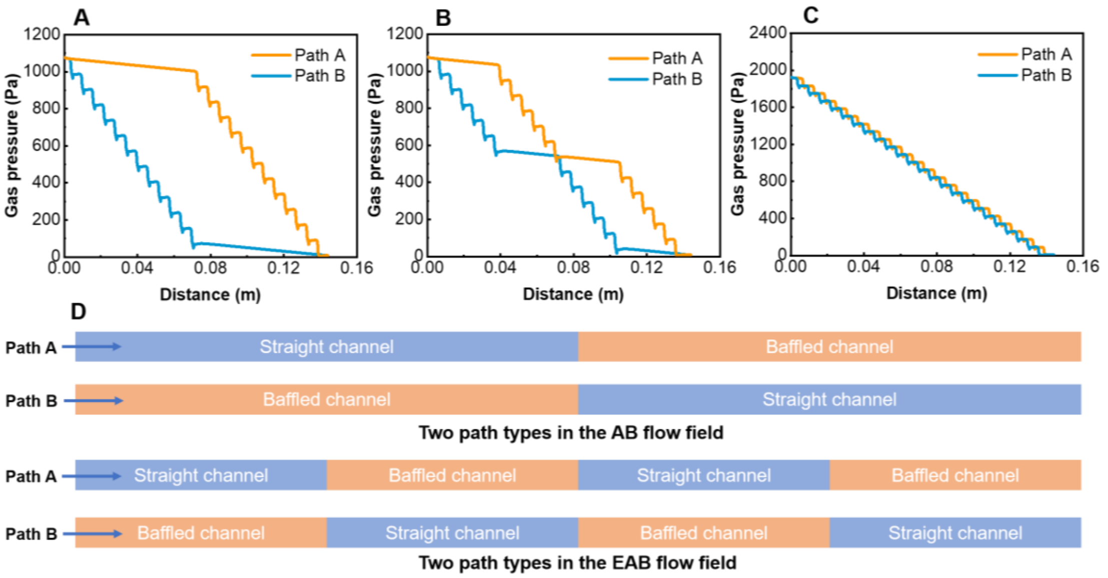

Figure S1. Simulation results of air pressure variation with distance in the two flow paths of (A) AB flow field, (B) EAB flow field, and (C) full baffled flow field. Operating conditions: current density (1.0 A cm^{-2}), RH (40%), ST (2.5), outlet back pressure (0 kPa), and operating temperature (343.15 K). (D) the schematics of structural unit arrangement for different flow fields.

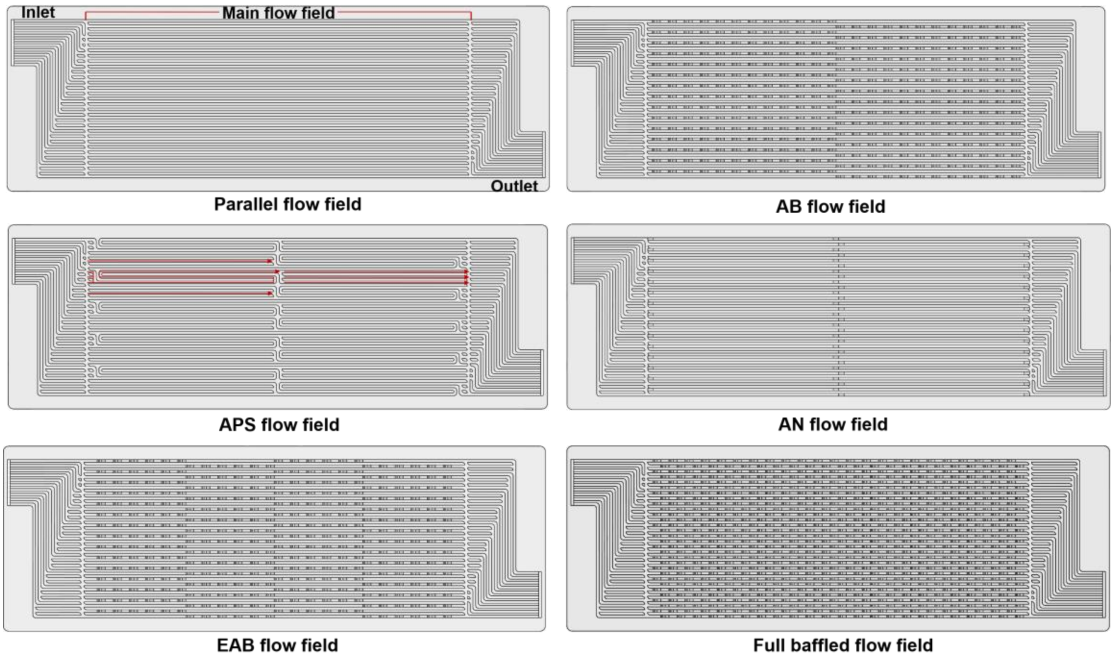

Figure S2. Schematics of the experimental flow fields

Table S1. Structural parameters of different cathode flow field designs

	Parallel flow field	AB flow field	AN flow field	APS flow field	Full baffled flow field	EAB flow field
Total area (cm^2)	106.2	106.2	106.2	106.2	106.2	106.2
Height of channels (mm)	1	1	1/0.5	1	1	1
Width of channels (mm)	1	1	1	1	1	1
Width of lands (mm)	1	1	1	1	1	1
Height of baffles (mm)		0.8			0.8	0.8
Length of baffles (mm)		3			3	3
Length of the leeward side of baffles (mm)		1.4			1.4	1.4
Length of the windward side of baffles (mm)		0.8			0.8	0.8
Total number of baffles of each channel		12			23	12
Interval of baffles along the channel (mm)		3			3	3

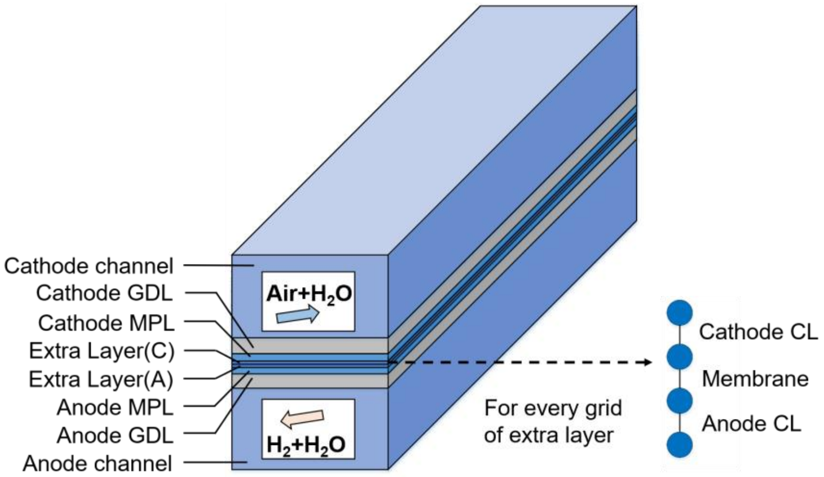

Figure S3. Computational domain of the CFD model[25, 26]

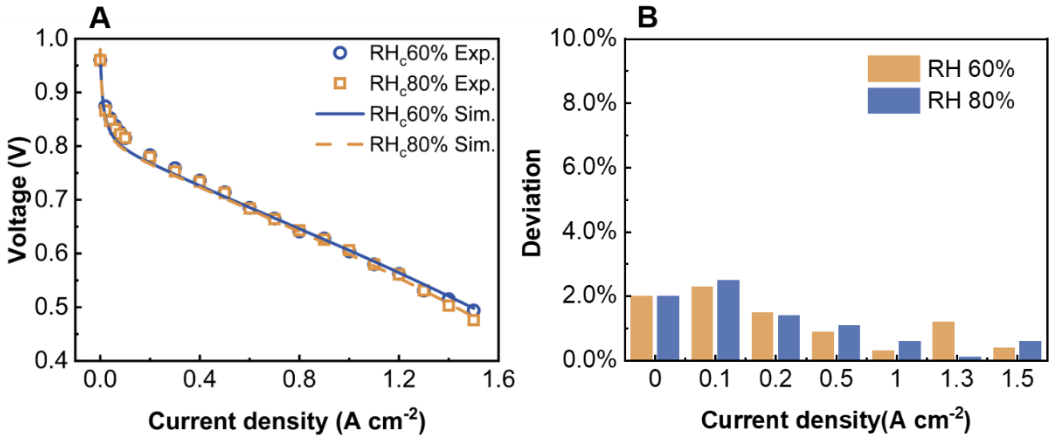

Figure S4. Comparison between the simulation results of CFD model and experimental data: (A) polarization curves; (B) deviation explanation. Operating conditions (anode/cathode): stoichiometric ratio (1.5/2.5), back total pressure (0/0 kPa), and operating temperature (343.15 K)

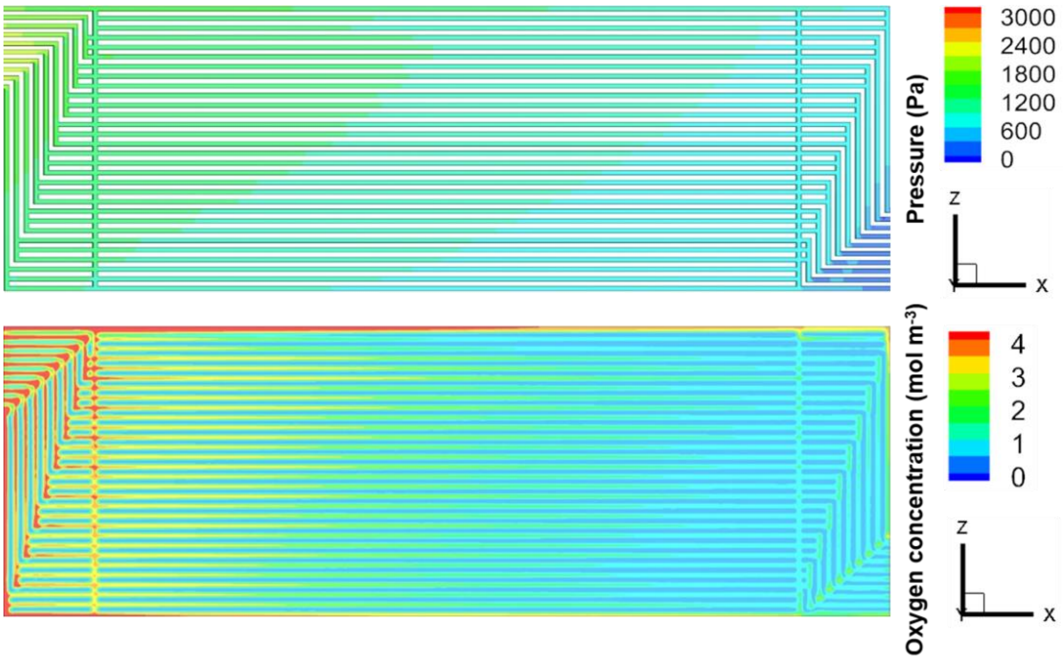

Figure S5. Pressure distribution in channels and oxygen concentration in the cathode CL of PEPFC assembled in a parallel flow field under RH_c 40% and ST_c 2.5

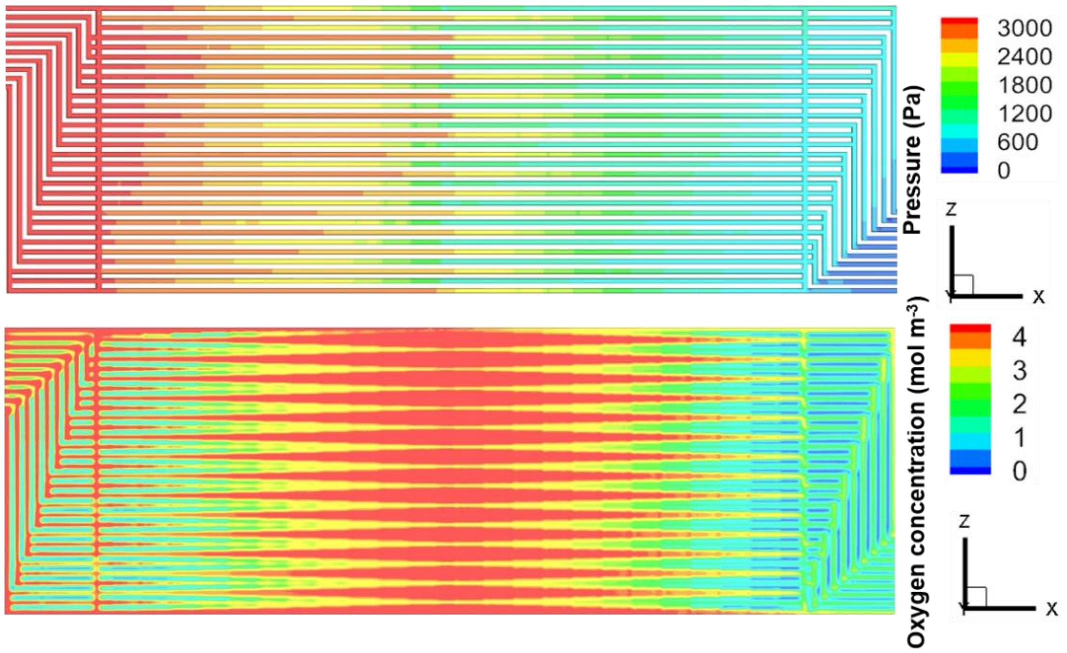

Figure S6. Pressure distribution in channels and oxygen concentration in the cathode CL of the PEPFC assembled AB flow field under RH_c 40% and ST_c 2.5

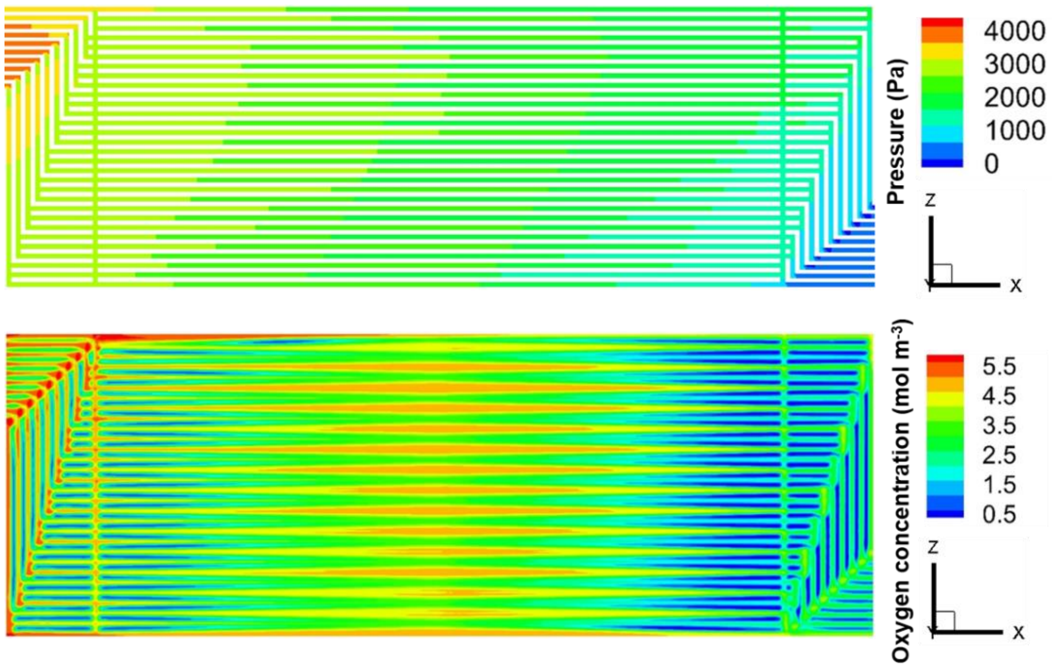

Figure S7. Pressure distribution in channels and oxygen concentration in the cathode CL of the PEPFC-assembled AN flow field under RH_c 40% and ST_c 3.5

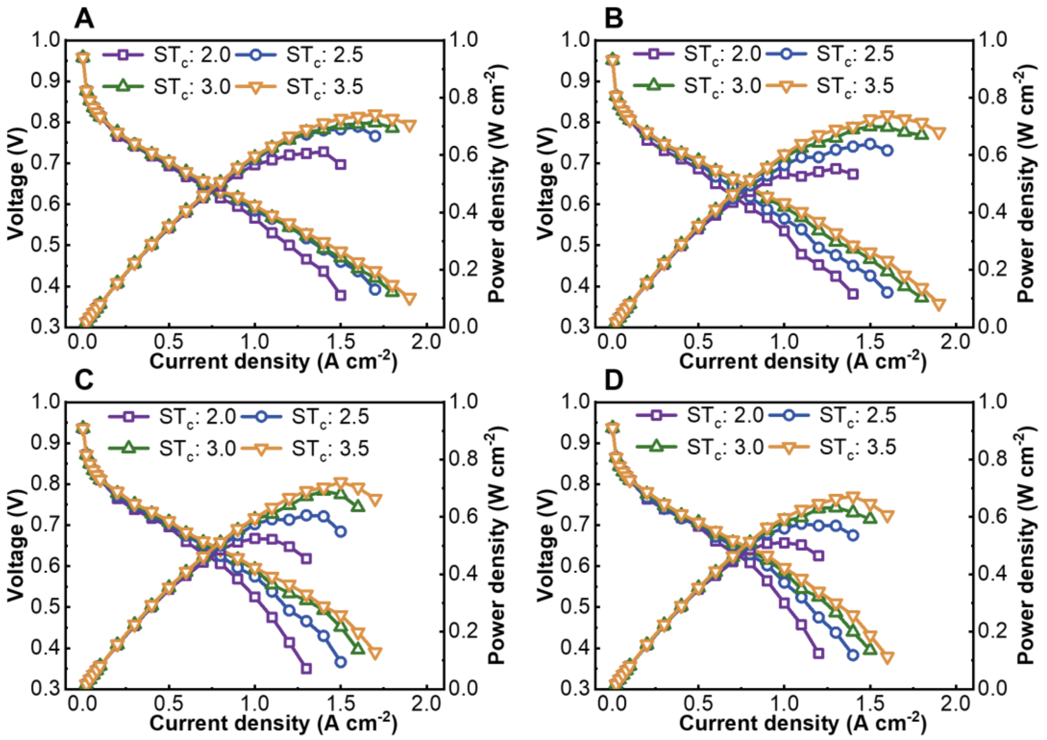

Figure S8. The experimental polarization curve of the PEMFC using a cathode APS flow field with an RH of the cathode inlet air of (A) 40%; (B) 60%; (C) 80% and (D) 100%

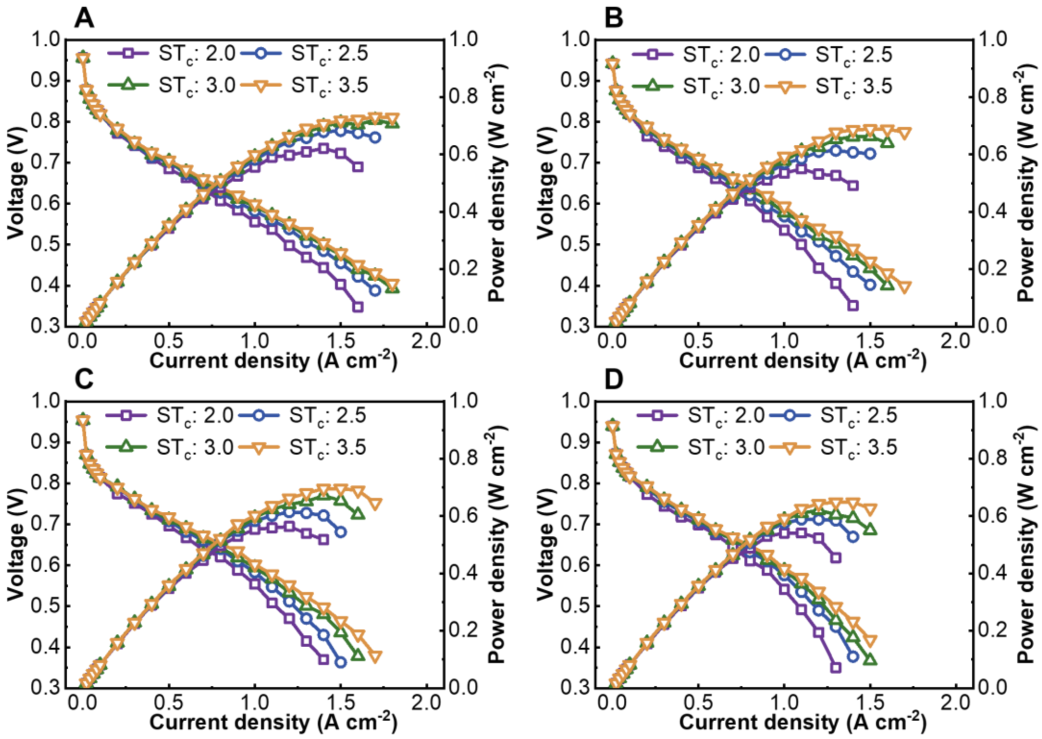

Figure S9. The experimental polarization curve of the PEMFC using a cathode AN flow field with an RH of the cathode inlet air of (A) 40%; (B) 60%; (C) 80% and (D) 100%

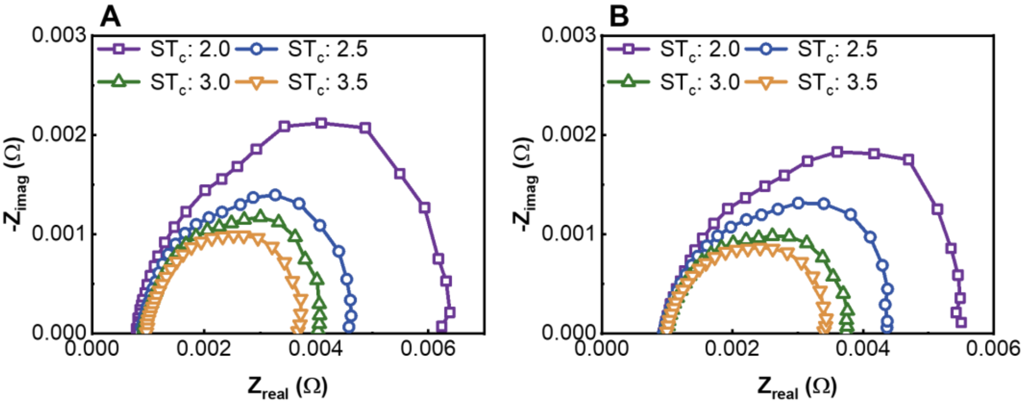

Figure S10. The experimental EIS test result under 1.0 A cm^{-2} of the PEMFC with an (A) APS flow field and (B) AN flow field at RH$_c$ 40%

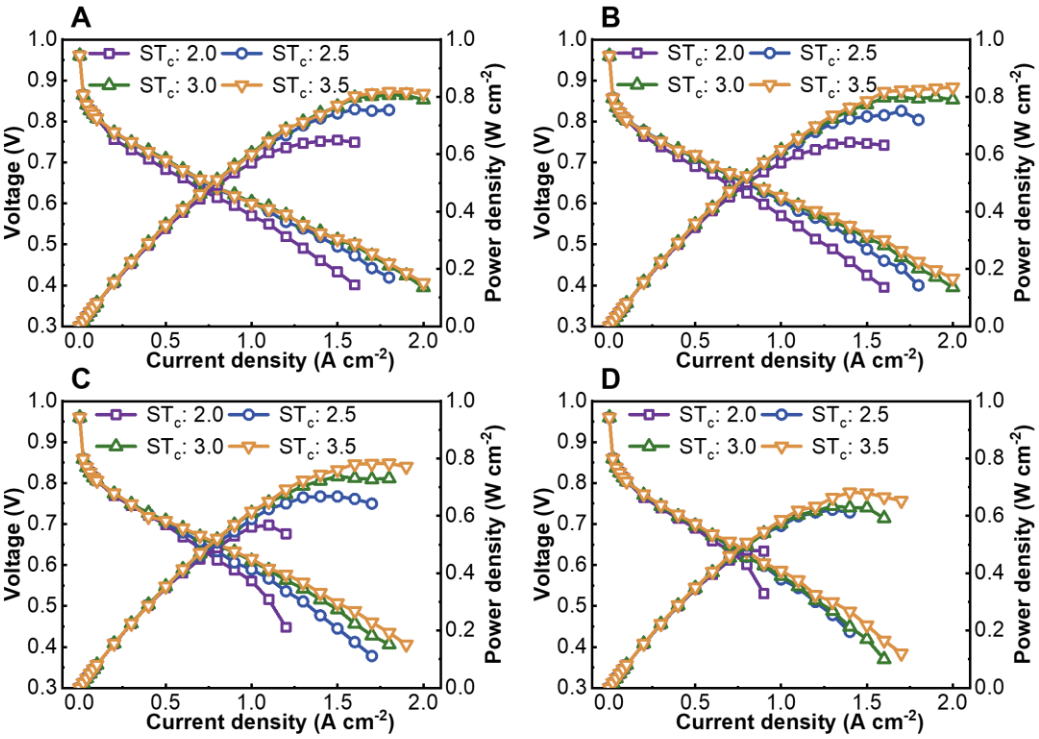

Figure S11. The experimental polarization curve of the PEMFC using a cathode full baffled flow field with an RH of the cathode inlet air of (A) 40%; (B) 60%; (C) 80% and (D) 100%

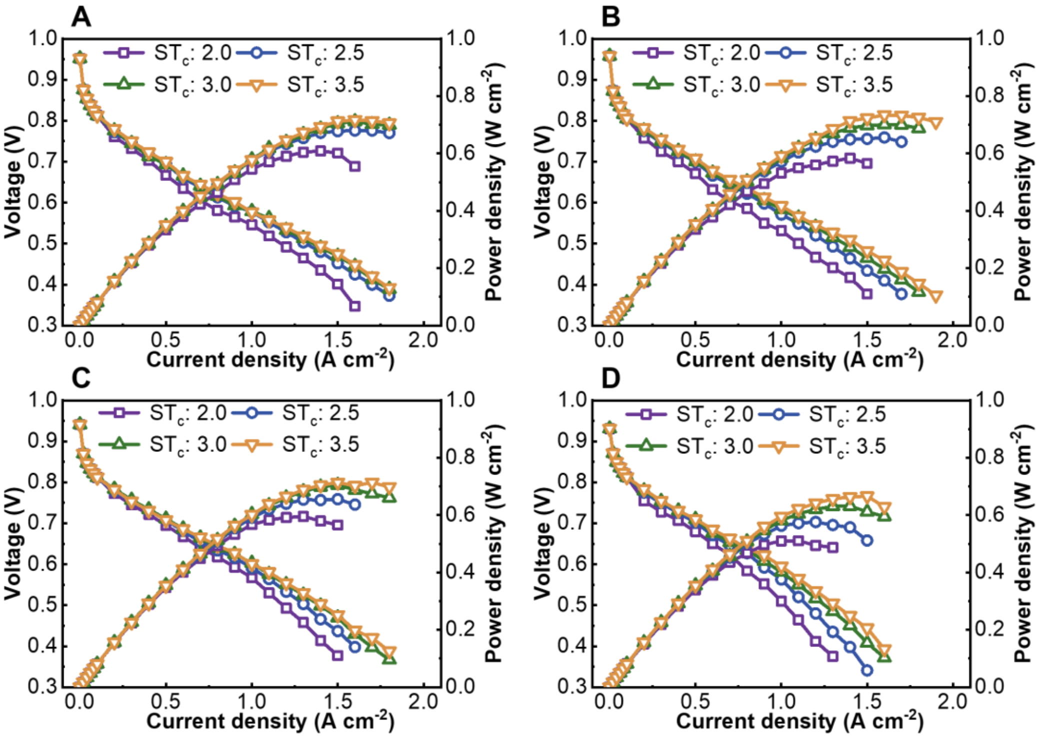

Figure S12. The experimental polarization curve of the PEMFC using a cathode EAB flow field with an RH of the cathode inlet air of (A) 40%; (B) 60%; (C) 80% and (D) 100%

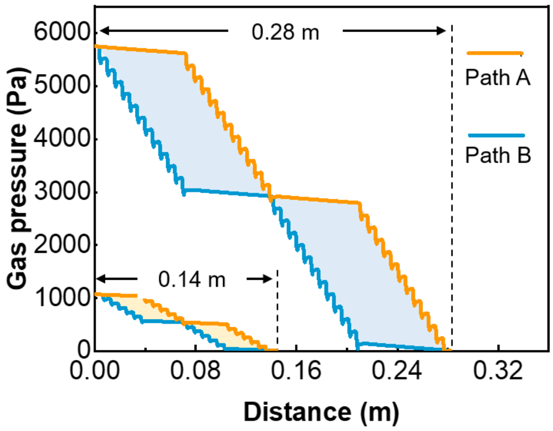

Figure S13. Simulation results of pressure difference between adjacent flow paths for the EAB flow fields with different lengths. Operating conditions: current density (1.0 A cm^{-2}), RH (40%), ST (2.5), outlet back pressure (0 kPa), and operating temperature (343.15 K)

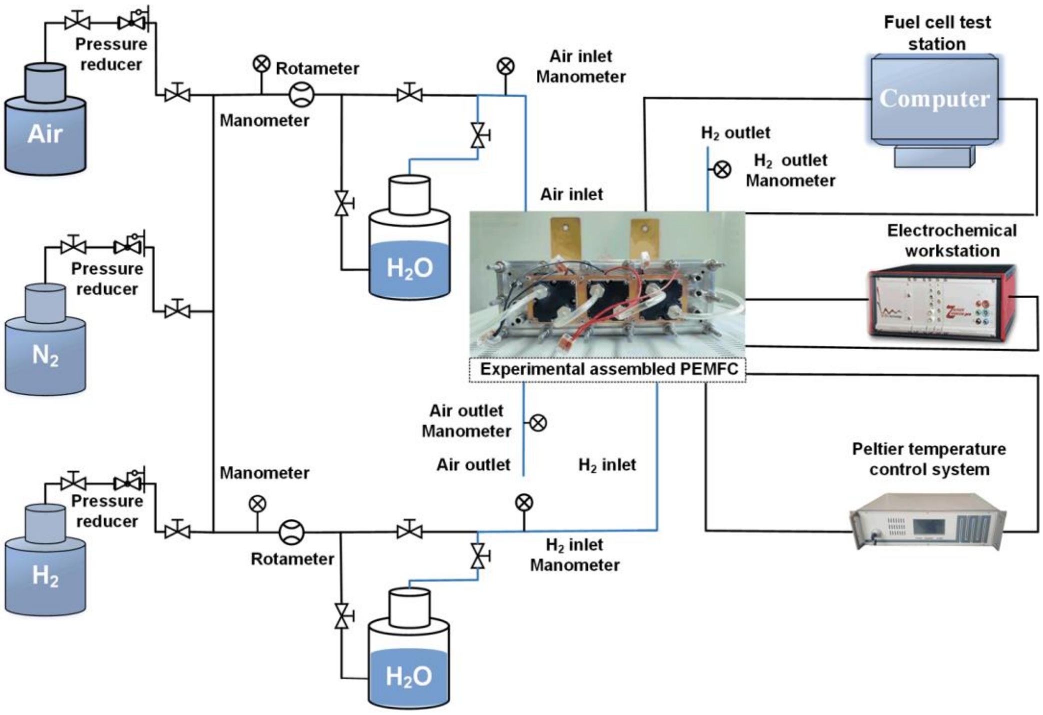

Figure S14. Schematic of the fuel cell test system

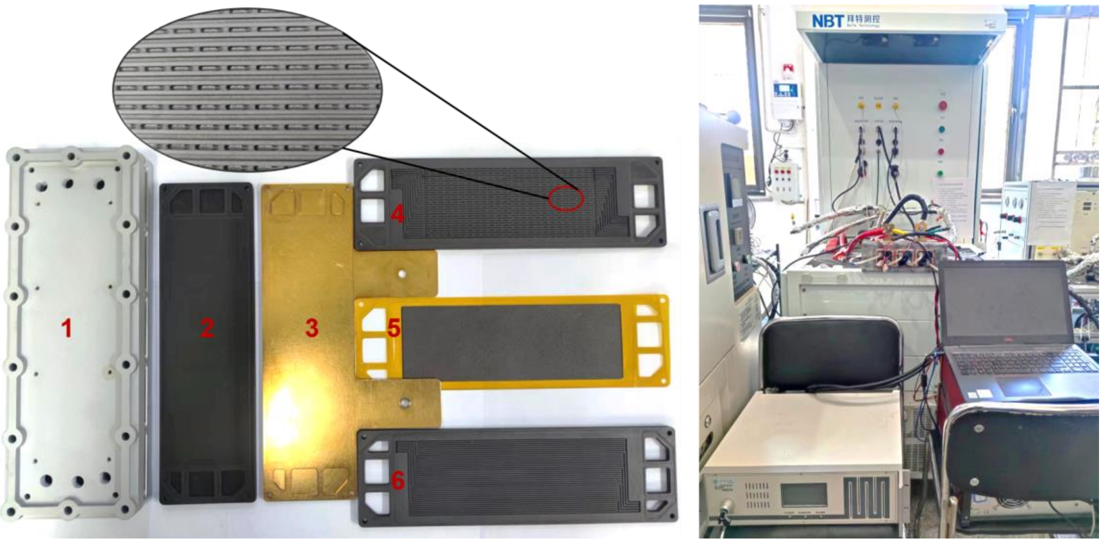

Figure S15. The components of the experimental PEMFC and the experimental system settings.

1-end plate; 2- insulated plate; 3- current collector plate; 4-flow field plate of cathode; 5-MEA; 6-flow field plate of anode.
